# Cultivated Meat and the Future of Food Systems: Promise, Progress, and Challenges

**DOI:** 10.1002/fsn3.71725

**Published:** 2026-04-07

**Authors:** Sungmin Kim, Keon‐Ha Park, Seong‐Jae Park, Yun‐Gwi Park, Sung‐Hwan Moon

**Affiliations:** ^1^ School of Cellular and Molecular Medicine University of Bristol Bristol UK; ^2^ Department of Animal Science and Technology Chung‐Ang University Anseong South Korea

**Keywords:** consumer acceptance, cultivated meat, food safety, market potential, nutritional value

## Abstract

Cultivated meat represents a substitute for traditional livestock farming through the external cultivation of animal cells. The technology is still in its infancy and requires continued research and development to achieve commercial viability. This analysis offers an overview of cultivated meat's current standing by examining its nutritional value and safety and comparing it with traditional meat options. The study examines both commercial viability and regulatory hurdles for market entry as well as consumer acceptance and psychological obstacles to adoption. The discussion encompasses food safety concerns, production costs, market opportunities, global regulatory approaches, and industry‐leading company trends in the cultivated meat field. The analysis presents key technological challenges and solutions while examining changes in consumer mindsets, besides sustainability and ethical issues, which remain crucial yet evolving aspects of cultivated meat development. The expanding global population has led to cultivated meat being recognized as a vital sustainable solution for future food security.

## Graphical abstract

An overview of cultivated meat as an emerging food‐production platform, contrasting the environmental burdens of conventional livestock with key biomanufacturing steps—from cell biopsy and proliferation to differentiation, scaffolding, and final product formation. The graphical abstract highlights anticipated benefits in sustainability, nutrition, and ethics, alongside major barriers to commercialization, including cost, public acceptance, regulatory challenges, and scale‐up.

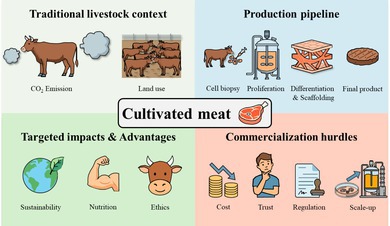

## 1 Introduction

The combination of global population changes and increased purchasing power in emerging markets alongside better living standards is driving meat consumption toward unsustainable levels (Press 2023). Modern food production systems face significant challenges due to rising demand for meat and dairy products, with implications for meat production through traditional livestock farming methods (Hong et al. 2021). The sustainability and ethical challenges of modern industrial livestock farming systems have proven extremely difficult to address successfully. Intensive animal agriculture sustains billions but is also linked to various global challenges (Godfray et al. 2018; Moran and Blair 2021). Notably, the livestock farming industry generates about 14.5% of worldwide greenhouse gas emissions, with cattle as the major contributor (Grossi et al. 2019).

Industrial meat production emerged as a strategy to feed an expanding human population yet resulted in substantial environmental damage (Rischer et al. 2020). Agriculture industry operations generate a large amount of greenhouse gases, which originate from both livestock enteric fermentation and manure emissions (Hilborn et al. 2018; Nugrahaeningtyas et al. 2024). The process of converting large areas of forest into grazing land intensifies global warming while leading to loss of biodiversity and destruction of ecosystems (Treich 2021; Tuomisto and de Mattos 2011). Additional complexity emerges from the public's concerns about animal welfare practices within intensive animal farming systems (Parlasca et al. 2023). A further issue associated with the livestock farming industry, and extending to the aquaculture industry, is the misuse of antibiotics to accelerate weight gain and control infection in food‐producing animals, which has become a significant threat to human health because of the growing antimicrobial resistance (O'Neill 2016; Van Boeckel et al. 2015).

**FIGURE 1:**
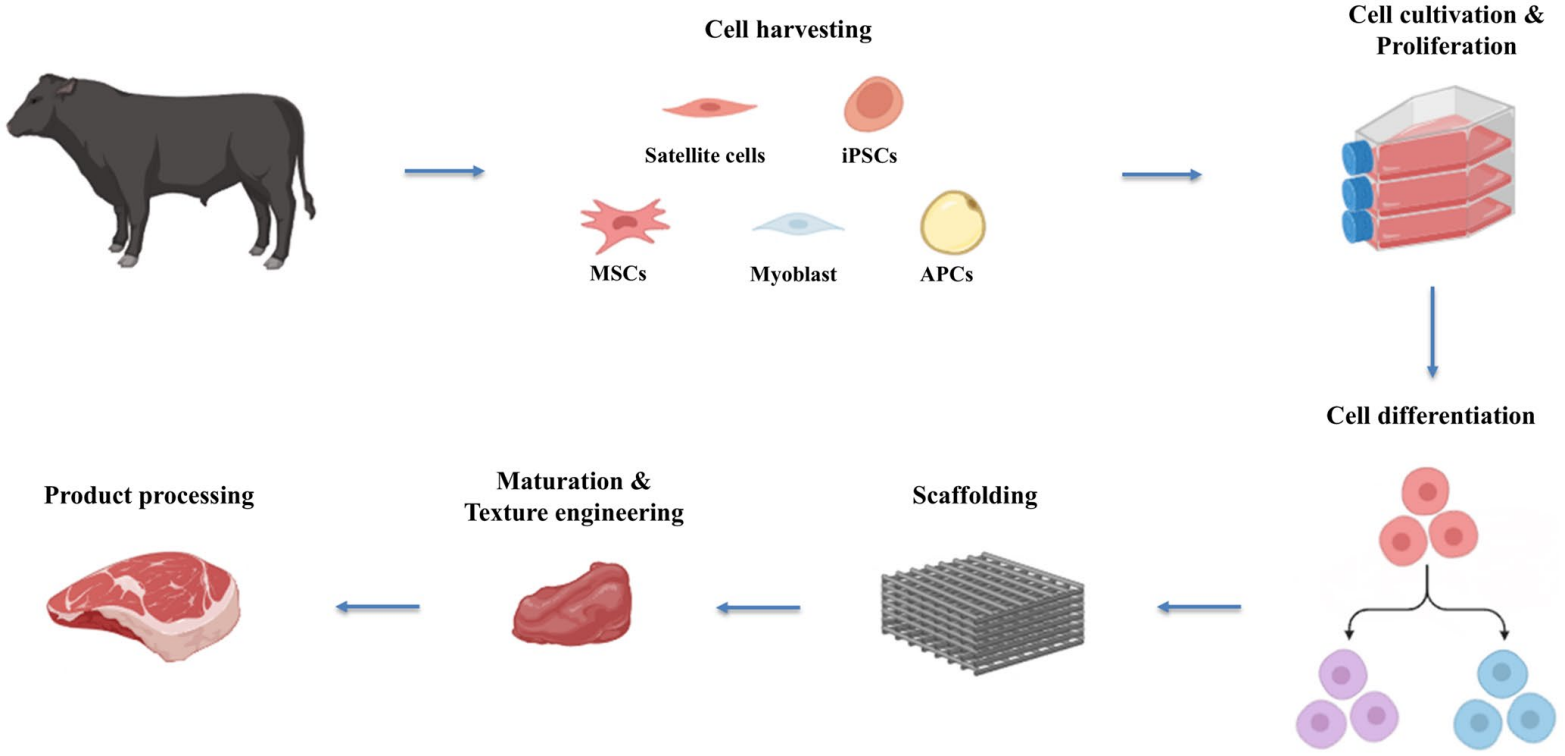
Conceptual diagram of the cultured meat production process. A schematic diagram of the cultured meat production process illustrates the harvesting of cells from cattle, followed by cell culture and growth, differentiation and scaffolding, maturation and texture engineering, and finally, product processing. Created with Biorender.com.

Due to these problems, there is a drive toward more sustainable and ethical methods for meat production (Biosphere Sustainable 2024). One possible alternative is the production of cultured meat using cellular agriculture, a technology that holds enormous promise for meeting these requirements and does not involve the slaughtering of livestock (Bhat and Hina Bhat 2011). There has been rapid progress in cultured meat production because of the convergence of biotechnology, tissue engineering, and food science which provides one means toward creating a sustainable food system (Figure 1) (Malila et al. 2024; Smetana et al. 2015).

From a myriad of possible strategies, laboratory‐grown or cell‐cultured meat is perhaps the most alluring. This approach introduces the possibility of reducing livestock farming and its related activities, with beneficial consequences for humans and the environment (Tullo et al. 2019). Ranching and butchering animals are replaced with growing their living cells under precise environmental conditions outside the body, which is termed cultivated meat, implying that meat is produced in a laboratory (Benny et al. 2022). This alternative method can help solve the problems encountered in conventional approaches to meat production. Raising cattle on extensive tracts of land requires large areas, often leading to deforestation (Steinfeld et al. 2006). Cell‐based agriculture will reduce land use and the ecological cost associated with livestock farming. Furthermore, the controlled environment for tissue culture would also help in improving the emission of greenhouse gases and reducing the overdependence on antibiotics (Lynch and Pierrehumbert 2019; Tuomisto and de Mattos 2011). One of the most compelling aspects of cultivated meat production is its potential to reduce animal suffering and promote animal welfare, addressing ethical issues associated with intensive animal agriculture and industrialized slaughterhouses (Schaefer and Savulescu 2014).

In this review, “cultivated meat” refers specifically to animal‐cell–derived tissue grown ex vivo through controlled proliferation and differentiation of myogenic, adipogenic, or stem‐cell lineages. This definition excludes plant‐based meat analogues and is conceptually distinct from hybrid products, which combine a small proportion of cultured cells with primarily plant‐derived matrices for structural or economic reasons. Throughout the manuscript, any discussion of nutritional, structural, or safety characteristics refers explicitly to fully cell‐based cultivated meat unless hybrid products are mentioned separately, as these categories differ markedly in composition, regulatory classification, and technological readiness.

In addition to providing an ethical and sustainable alternative to conventional meat production, cultivated meat also holds considerable potential for transforming other traits of meat, such as its nutrients (Hocquette et al. 2025). With precise control over the surrounding environment during its production, it is possible to manipulate the nutritional qualities of cultivated meat (Ching et al. 2022). This would facilitate the manufacture of meat with a superb content of fat, a higher level of protein, or even greater amounts of other nutrients than its conventional counterparts (Anzani et al. 2020).

Despite its potential, cultivated meat faces significant hurdles, particularly its high production costs, which limit its commercial viability (Bryant et al. 2019; Stephens et al. 2018). Perhaps the largest concern is the relatively high cost of cell‐based beef production, which prevents it from being commercially feasible (Drouillard 2018). It should also be noted that as the development of cultivated meat is still at the laboratory scale, the transition to large‐scale meat production will require advances in cell culture techniques and biomanufacturing methods. Increasing production efficiency, cell growth, and differentiation and creating an appealing meat texture are most critical (Choudhury et al. 2020). While the technology will revolutionize the food industry, its acceptance within society will likely be slow due to the unwillingness to compromise on traditional farming practices (Abiri et al. 2023). With the advancements in techniques for producing cultivated meat, the regulations pertaining to production, labeling, consumer safety, and transparency are expanding but require greater attention as, like other technologies, there exist psychological barriers to accepting its “naturalness” (Siegrist et al. 2018; Verbeke, Sans, and Van Loo 2015). Consumer acceptance remains critical, as cultural and psychological factors heavily influence the adoption of novel food (Hartmann and Siegrist 2017; Wilks and Phillips 2017).

Although cultivated meat has been widely promoted as a sustainable alternative to conventional livestock, it is important to note that most current market offerings are hybrid products—primarily composed of plant‐based matrices supplemented with small amounts of cultured animal cells. These products serve more as transitional innovations rather than full replacements for conventional meat, and their nutritional and structural profiles do not yet match those of traditional meat. Therefore, evaluating cultivated meat technologies must include a clear distinction between theoretical capabilities and commercial realities (Alam et al. 2024).

Recent institutional and foresight reports have also explored the future trajectories of cultivated meat within sustainable food systems and policy frameworks (Chan 2024). Building upon these broader perspectives, we will evaluate the entirety of cultivated meat, from its nutritional safety to economic regulations, by examining the cost of production and analyzing the market and international regulations. This paper concludes with our opinion on the prospects and challenges of the technology, user behavior modification techniques, and concerns regarding sustainability and ethics. As alluded to above, while cultivated meat presents significant potential, it is also accompanied by considerable technological and regulatory challenges.

## 2 Factors Associated With Nutrition and Safety: An Evaluation of Meat Structure and Safety of Cultivated Meat

Consumers can expect that the food they eat is safe, healthy, and wholesome (Gardner 1993). As cultivated meat is a novel food, for it to succeed as an alternative meat market, the industry must assuage potential safety concerns around a novel food while delivering a nutritious product at a comparative price to traditional meat products (Gu et al. 2023). This section compares cultivated and traditional meat, assesses the safety issues related to cultivated meat production, and addresses consumer concerns to facilitate informed choices (Pakseresht et al. 2022).

Traditional meat structure is routinely assessed using established analytical techniques such as Texture Profile Analysis (TPA) and Warner–Bratzler shear force measurements to quantify hardness, cohesiveness, springiness, and tenderness. These instrumental methods are longstanding standards in meat science and are widely used to characterize structural integrity and consumer‐relevant texture attributes in conventional meat products (Schmid et al. 2022). They can also be directly applied to cultivated meat, as scaffold‐based or assembled tissues similarly require evaluation of fiber alignment, moisture retention, connective tissue organization, and post‐processing texture. Microscopic and histological approaches—including scanning electron microscopy (SEM), transmission electron microscopy (TEM), and myofiber staining—likewise remain applicable for examining microstructural development in cultured tissues (Chakravorty and Das 2024).

From a food safety perspective, conventional assessment frameworks readily translate to cultivated meat. Microbiological monitoring—including aerobic plate counts, pathogen detection (e.g., *Salmonella*, *Listeria*), mycoplasma screening, and chemical residue testing—forms the foundation of hazard identification and quality control in meat processing (Sofos 2008). Although cultivated meat benefits from a closed, aseptic bioprocess, rigorous Hazard Analysis and Critical Control Point (HACCP)–aligned monitoring remains essential to ensure contamination prevention and safe downstream handling (Powell et al. 2025). These parallels demonstrate that many established analytical and safety assessment tools from traditional meat science remain directly relevant and applicable to the evaluation of cultivated meat.

### 2.1 Nutritional Value Comparison (Traditional Meat vs. Cultivated Meat): “Replicating and Innovating on the Blueprint of Nature”

Similar to traditional meat, cultivated meat consists of muscle, fat, connective tissues, and bones. As a result, it is capable of replicating the nutritional profile of traditional meat, including its high content of protein, iron, vitamins, and other vital micronutrients (Table 1) (U.S. Food and Drug Administration (FDA) 2023; Fraeye et al. 2020; Lee et al. 2024; U.S. Department of Agriculture and Agricultural Research Service 2023). However, unlike traditional animal agriculture, where breed and climate affect nutrition, cell culture technology in meat production has no such restrictions. These factors can be fine‐tuned for optimization. For example, cultivated meat can be designed to contain higher levels of unsaturated fatty acids and lower levels of saturated fatty acids to mitigate risks of cardiovascular diseases (Willett et al. 2019). The ratio of omega‐6 to omega‐3 fatty acids can also be changed for improved cardiovascular health and inflammation reduction (Calder 2015). In addition to fat adjustment, cultivated meat can be supplemented with other nutrients deficient in conventional meat (Kang et al. 2024). To prevent anemia, the product can be fortified with iron (Hurrell and Egli 2010). Likewise, more vitamins and minerals can be included to further enhance the nutritional value. The protein percentage can be controlled during the process, which is important as the daily consumption of food from animal and fish sources is associated with enhanced nutritional status (Antonio et al. 2020). Finally, the amino acid profile could be designed to contain all the essential amino acids (Wu 2016).

**TABLE 1:** Nutritional composition of cultivated vs. conventional meat (per 100 g) composition (per 100 g) of raw beef, raw assembled cultured meat (ACM), USDA skinless chicken, and cultured poultry meat (CPM) produced with serum‐containing and serum‐free media.

Nutrient (g)	Raw beef	Raw ACM	USDA skinless chicken	Serum‐containing CPM	Serum‐free CPM
Carbohydrate	7.97	3.00	0.00	2.18	0.00
Protein	19.88	10.71	22.50	14.53	18.02
Saturated Fat	3.29	0.12	0.35	0.60	0.53
Unsaturated Fat	3.41	0.017	0.67	0.97	1.27
Total Fat	6.70	0.137	1.02	1.57	1.80

*Note:* Data were compiled from published reports and studies (U.S. Food and Drug Administration (FDA) 2023; Fraeye et al. 2020; Lee et al. 2024; U.S. Food and Drug Administration 2023).

Abbreviations: ACM, Assembled cultured meat; CPM, Cultured poultry meat.

Standard nutritional assessment methods from meat science are directly applicable to cultivated meat. Protein quantification can be performed using Kjeldahl or Dumas nitrogen determination, while amino acid composition is typically analyzed through HPLC or LC–MS. Fatty‐acid and lipid class profiles are evaluated via GC–MS, consistent with traditional meat chemistry workflows (Schmid et al. 2022). Protein quality is assessed using the Protein Digestibility‐Corrected Amino Acid Score (PDCAAS), a widely accepted metric of amino acid availability and digestibility in food proteins (Hoffman and Falvo 2004). These results are contextualized against WHO/FAO recommended daily protein intake guidelines to assess the nutritional value of cultivated meat in the human diet (WHO 2007).

In addition to amino‐acid composition, protein quality and bioavailability depend on the structural organization of the muscle matrix. Conventional meats typically display high Digestible Indispensable Amino Acid Scores (DIAAS), often exceeding 100 for beef and pork, reflecting superior ileal digestibility and essential‐amino acid availability (Bailey et al. 2020). Early analyses of cultured muscle fibers show that, when myotube alignment and sarcomere maturation are achieved through mechanical or biochemical stimulation, digestibility approximates that of conventional meat. Conversely, tissues produced with minimal stimulation may exhibit reduced proteolytic accessibility due to immature matrix formation (Fraeye et al. 2020). The FAO's (2013) expert report highlights DIAAS as a more accurate indicator of absorbed amino‐acid content than PDCAAS and recommends its use for future protein quality comparisons between cultivated and traditional meats (FAO 2013). As scaffold engineering and maturation strategies improve, the bioavailability of nutrients in cultivated meat is expected to further converge with that of conventional muscle‐based foods.

The choice of cell lines, growth media, and production methods can influence the composition of cultivated meat so that the product delivers a similar or better nutritional profile than conventional meat (Gaydhane et al. 2018). Hence, comprehensive nutritional studies of different cultivated meat products are needed to ascertain that they fulfill all standards and describe their nutritional profile fully (Piantino et al. 2025).

The nutritional characteristics of cultivated meat are strongly influenced by both cellular and bioprocess variables. The intrinsic properties of the chosen cell line—such as fiber‐type specification, myogenic differentiation capacity, and adipogenic potential—determine the balance of myofibrillar proteins, intramuscular fat, and connective tissue components. Growth‐media composition further modulates fatty acid profiles, micronutrient incorporation, and intracellular metabolite accumulation, and has been shown to significantly impact the nutritional and sensory attributes of engineered tissues (Piantino et al. 2025). Process parameters—including scaffold architecture, mechanical stimulation, and bioreactor culture conditions—can enhance myofiber alignment, collagen deposition, and lipid distribution, collectively shaping the final macro‐ and micronutrient profile of the cultivated product (Stout et al. 2023).

### 2.2 Considerations on Cell Line Origin and Market Realities

Although the nutritional makeup of farmed meat may be comparable to or even better than that of conventional meat, it is important to place these claims within the constraints of current technology and the market (Broucke et al. 2023). The immortalized chicken B‐cell line DT‐40, which was initially created for laboratory use rather than human consumption, is used to create a sizable portion of cell‐based meat prototypes (Winding and Berchtold 2001). Although these cells are helpful for scalability in experiments, they do not accurately reflect the nutritional and structural characteristics of skeletal muscle tissue, especially with regard to the amount of protein and the makeup of amino acids.

In addition to DT40, several cell types are well‐established in cultivated meat research and development. Primary bovine and porcine satellite cells and myoblasts closely resemble conventional muscle tissue, whereas pluripotent stem cell–derived myogenic and adipogenic progenitors enable scalable and renewable cell sourcing (Piantino et al. 2025). Immortalized and model cell lines such as C2C12 (myogenic) and 3T3‐L1 (adipogenic) also serve as robust platforms for process optimization and early‐stage prototyping (Stout et al. 2023). Furthermore, some cell types exhibit spontaneous differentiation or fusion when exposed to specific cues, which can reduce the need for growth‐factor supplementation but require careful control to ensure batch uniformity and regulatory compliance.

Additionally, the majority of cultivated meat products that are on the market or in the process of being developed are hybrid formulations that incorporate a small percentage of cultured cells into a matrix derived from plants (Rubio et al. 2020). Because of their limited cellular content, these hybrid products frequently lack the complete nutritional equivalency of conventional animal meat, despite being primarily designed to mimic the appearance and taste of meat. Any nutritional claims about cultivated meat must therefore make a distinction between theoretical potential, which is predicated on fully tissue‐engineered muscle structures, and actual commercial products, which might not meet these requirements (Post 2012).

The need for clear labeling, thorough compositional analyses, and uniform nutritional profiling across farmed meat products is highlighted by this disparity. Only by taking such steps can legislators create suitable regulatory frameworks and consumers make educated decisions.

Hybrid cultivated–plant matrices have distinct nutritional and structural consequences. Plant‐derived proteins in these systems can dilute essential amino acid density and introduce antinutritional factors—such as phytates and protease inhibitors—that reduce protein digestibility and amino acid bioavailability, as seen in plant‐based analogues (Bogueva and McClements 2023). Replacing animal‐derived phospholipids with plant oils changes the saturated‐to‐unsaturated fat ratio and may affect oxidative stability and lipid‐related nutritional outcomes (Yan et al. 2024). Structurally, hybrid products often exhibit softer textures and lower water‐holding capacity because plant matrices limit myotube alignment, preventing the formation of anisotropic, muscle‐like fibers characteristic of conventional meat (Song et al. 2026). Recent analyses indicate that hybrid systems continue to face significant challenges in achieving meat‐like technofunctional properties, underscoring the need to classify them separately from fully tissue‐engineered cultivated meat in both technological and nutritional contexts (Kaplan and McClements 2025).

### 2.3 Food Safety: The Issues of Maintaining the Hygiene and Quality of Cultured Meat Products

It is important that cultivated meat adheres to all food regulations and, like any other food product, must comply with strict food safety standards (Cai et al. 2024). Although some contamination risks are reduced during cell culture, other safety issues require active remediation. Microbiological contaminants (bacteria, viruses, fungi) pose the greatest risk (Chawla et al. 2023). Some of these safety measures can include the adoption of enhanced sterile protocols, more intense quality assurance efforts, and extensive use of Hazard Analysis and Critical Control Point (HACCP) systems (Sofos 2008). Allowing access to active industrial production processes may help eliminate contamination and prevent its recurrence. The growth factors used in cultivated meat production promote cell growth and differentiation; however, the end product must be of high purity and consistent quality (Ahmad et al. 2023). While initial studies do not indicate any toxicity, long‐term safety studies of cultivated meat are critical (Ben‐Arye et al. 2020). To develop consumer confidence, all stakeholders dealing with this technology must ensure proper communication and information sharing and guarantee that the correct regulations are in place.

Long‐term safety evaluation of cultivated meat extends well beyond routine microbiological checks and now aligns with the broader framework used for novel foods. Regulatory agencies, including the European Food Safety Authority (EFSA), emphasize the need for structured toxicological studies—particularly sub‐chronic and chronic exposure assessments—to determine potential cumulative risks associated with repeated consumption (EFSA Panel on Dietetic Products et al. 2021). Allergenicity is another important consideration, as scaffold materials, serum replacements, and culture‐derived byproducts can introduce or alter protein epitopes. To address these risks, the EFSA and the FDA recommend a combination of sequence‐homology screening, in vitro digestibility assays, and characterization of any potentially novel peptides that may arise during production (EFSA Panel on Dietetic Products et al. 2016; U.S. Food and Drug Administration 2023). In parallel, agencies increasingly highlight the importance of metabolic and nutritional safety assessments, including the evaluation of lipid oxidation products, amino‐acid turnover, and other bioactive compounds generated during culture or processing. These measures help ensure that the metabolic behavior of cultivated tissues is well understood and that any differences from conventional meat are identified early (EFSA Panel on Dietetic Products et al. 2021; U.S. Food and Drug Administration 2023).

Although regulatory frameworks in the European Union (EU), the United States, and Singapore provide a solid foundation for evaluating cultivated‐meat products, many authorities acknowledge that additional data will be needed as production scales up. This includes more comprehensive evidence on long‐term metabolic effects, potential immune responses to culture‐derived components, and the fate of scaffold‐ or media‐related residues during digestion. Such considerations reflect a broader regulatory trajectory: while current guidelines are appropriate for early‐stage assessments, full industrial deployment will require a more granular understanding of how cultured tissue products behave physiologically over time (EFSA Panel on Dietetic Products et al. 2021; Singapore Food Agency 2025).

### 2.4 Deep‐Rooted Psychological Problems and the Acceptance of New Technology: Unfulfilled Acceptance and Innovations

Another major challenge is overcoming consumer inertia, which is influenced by psychological factors and a lack of information. The perception of cultivated meat as unnatural is one of the principal factors (Wilks et al. 2020). Cellular agriculture, being a novel and technologically advanced concept, may not resonate with many people due to its perceived unnaturalness and complexity. Such worries are exacerbated by feelings of unfamiliarity, where measures that alter technology and safety are to some degree a concern about how safe these measures are. Extreme anxiety about potential long‐term health consequences poses yet another barrier. Attempts to confront the psychological obstacles to realizing the benefits of cultivated meat must be made through constructive dialogue. Transparency concerning the safety and regulatory frameworks as well as the level of enrichment of these products must be ensured (Mancini and Antonioli 2019; Verbeke, Sans, and Van Loo 2015).

Meat consumption is also affected by sociocultural and religious factors. Acceptance needs to be strategized at a completely different level. Public education campaigns can aid in the acceptance of unfamiliar technology. Any technology that claims reduced carbon emission and enhanced animal care will garner the support of more socially responsible consumers (Bryant and Barnett 2018; Hartmann and Siegrist 2017). Most importantly, the product's flavor and mouthfeel will greatly influence its acceptance among consumers (Singh et al. 2022). To benchmark cultivated meat against conventional meat, internal taste tests must be performed alongside conventional meat.

## 3 Economic and Regulatory Landscape: Implementing Commercialization and Market Integration

Thorough research is needed to transition cultivated meat from the laboratory to commercialization successfully. This section discusses the categorization of cultivated meat as a credible food product within mainstream markets (Bekker et al. 2021). It aims to analyze the market and industry of cultivated meat as well as investigate the phenomena affecting production costs (Garrison et al. 2022). It also looks into the changing international regulatory environment, paying attention to the different regions' approaches to regulation, as well as the fundamental issues of consumer protection versus promotion of creativity (Arslan et al. 2021). It concludes with an overview of the most important companies in the cultivated meat industry, which paints a picture of the vibrancy of this emerging industry (Dueñas‐Ocampo et al. 2023).

### 3.1 Production Costs and Market Potential: Bridging the Gap: Realizing the Commercial Potential of Cultivated Meat

The market for cultivated meat faces a significant challenge: high production costs (Kirsch et al. 2023; O'Neill et al. 2021). Due to the price of growth media, growth factors, and specialized equipment, the cost of growing cultivated meat is significantly higher than traditional meat production (Humbird 2021). However, optimizing cell culture processes, developing more cost‐effective growth media, and tackling the scaling problem hold great potential to overcome this hurdle (O'Neill et al. 2021; Post et al. 2020).

While the high cost of cultivated meat is largely driven by growth media, growth factors, and specialized equipment, ongoing research is actively addressing these challenges (Humbird 2021). For instance, plant‐based media are being developed to replace costly animal‐derived components (Bakhsh et al. 2025), and more affordable alternatives for growth factors are under exploration to improve cost‐efficiency (Bodiou et al. 2020).

Despite the current high unit cost, the potential for mass production renders cultivated meat a promising target for investment. In order to utilize an economy of scales, bioreactor and automated plant infrastructure are needed (Humbird 2021). A few companies are starting to invest in such infrastructure in order to lower the production costs while also increasing productivity (Stephens et al. 2018).

**FIGURE 2:**
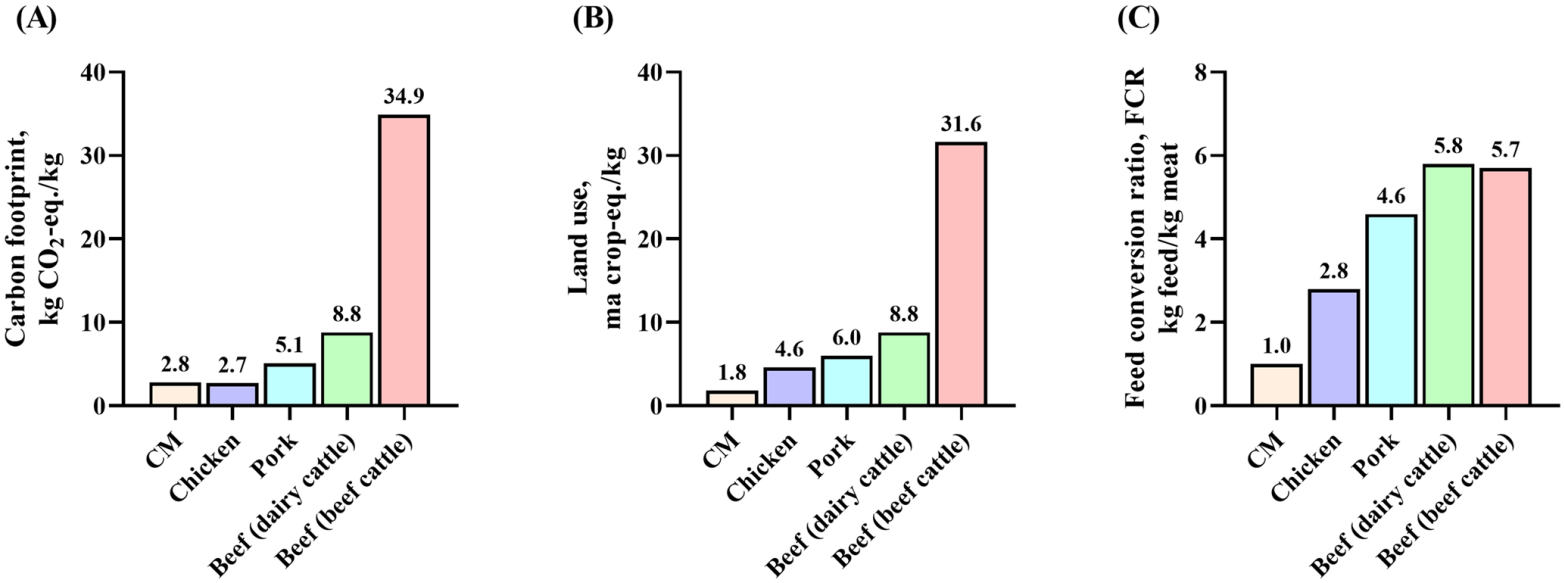
Environmental impact of cultivated meat vs. conventional meat. (A) Greenhouse gas emissions: Conventional livestock farming emits significantly more greenhouse gases than cultivated meat production. Data from (Sinke et al. 2023). (B) Land use: Cultivated meat production requires less land compared to conventional beef, pork, and chicken farming. Data from (Sinke and Ingrid 2021). (C) Feed conversion ratio, kg feed/kg meat: Cultivated meat demonstrates higher feed efficiency than conventional livestock. CO_2_‐eq: Carbon dioxide equivalent; CM: Cultivated meat. Data from (Sinke et al. 2023).

Despite the high production costs, the market prospect for cultivated meat seems positive and is likely to improve as consumer awareness of its ethical and environmental benefits increases (Figure 2) (Bryant 2020; Sinke et al. 2023; Verbeke, Marcu, et al. 2015). As the population grows globally and the demand for meat increases, the potential market opportunities also improve (Tuomisto and de Mattos 2011). The realization of this forecast is however contingent upon reduction in production costs, achieving traditional meat price levels, and securing required permits (Ching et al. 2022). Several techno‐economic analyses (TEAs) have reported projected production costs for cultivated meat (Garrison et al. 2022; Humbird 2021; Robert Vergeer and Ingrid 2021). More recent industry reports, such as the 2025 Lever VC analysis, cite notably lower company‐reported cost ranges (Lever VC 2025). These estimates are summarized in Table 2.

**TABLE 2:** Summary of key techno‐economic analysis (TEA) estimates for cultivated meat production.

Study/source	Year	Scale/assumptions	Estimated cost (USD/kg)
Humbird (Open Philanthropy TEA)	2021	Conceptual facilities; fed‐batch & perfusion	$37–51/kg
Garrison et al. (JAFR)	2022	Large‐scale facility (540,000 kg/year)	$63/kg
CE Delft TEA	2021	2030 full‐scale scenarios	Scenario‐dependent
Lever VC report	2025	Company‐reported data	$10–15/kg (claimed)

*Note:* Data were compiled from published reports and industry analyses (Garrison et al. 2022; Humbird 2021; Lever VC 2025; Robert Vergeer and Ingrid 2021).

Although the long‐term goal of cultivated meat is to use advanced cellular agriculture to produce whole cuts of animal tissue, the commercial products that are currently on the market greatly deviate from this ideal (Rubio et al. 2020). The majority of the farmed meat products currently sold in a few markets, including Singapore and the US, are hybrid products made primarily of plant‐based ingredients with a small amount of cultured animal cells (Alam et al. 2024).

Usually, these formulations are designed to mimic the flavor, appearance, and mouthfeel of meat while lowering production costs and working around current regulatory frameworks. However, rather than making a substantial contribution to nutritional or structural authenticity, the actual cellular content in these products is frequently negligible and only serves as a symbolic inclusion to validate the cultivated label (Pakseresht et al. 2022).

Because of this strategy, some industry critics contend that the cultivated meat that is currently available may be “cultured for show” rather than being functionally equivalent to traditional meat. In order to distinguish between aspirational narratives and realistic market outputs, this divergence must be taken into consideration in both public perception and scholarly discussions surrounding cultivated meat. Therefore, the percentage of cultured material, the function of plant‐based scaffolds, and their effects on labeling, nutrition, and consumer trust should all be made clear in future studies and policy discussions.

Several TEAs have evaluated the projected production costs of cultivated meat. Humbird estimated costs between USD 37 and 51 per kg, depending on media purity requirements and bioreactor configuration (Humbird 2021). Garrison et al. (2022) projected a cost of USD 63 per kg for a large‐scale facility with a capacity of 540,000 kg/year. In addition, CE Delft's 2021 TEA indicated that substantial reductions in media costs, improvements in cell density, and integration of renewable energy could bring costs closer to commercial viability by 2030 (Robert Vergeer and Ingrid 2021). Collectively, these assessments highlight both the current economic barriers and the potential pathways for cost convergence with conventional meat.

### 3.2 The Global Regulatory Terrain Scan: Striking a Balance Between Safety and Innovation

**FIGURE 3:**
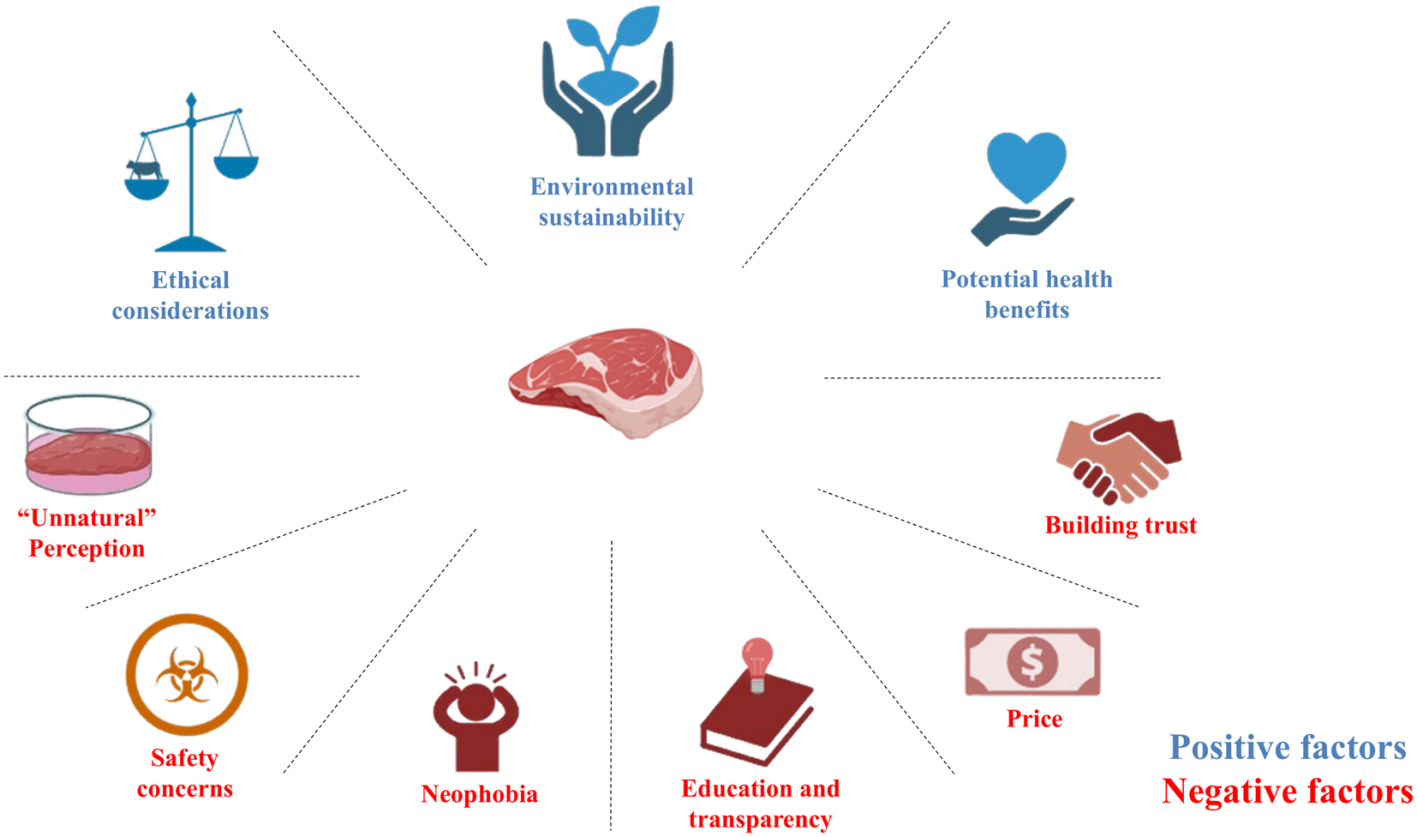
Consumer acceptance factors. The positive factors, arranged from left to right, consist of ethical considerations, environmental sustainability, and potential health benefits. In contrast, the negative factors encompass safety concerns, high price, and the challenge of building consumer trust. Created with Biorender.com.

New regulatory changes are emerging for cultivated meat globally, but the approaches vary across regions. Some governments have created positive laws on cultivated meat, while others are still developing the laws (Stephens et al. 2018). The primary regulatory issues are identified as food safety, product labeling, and consumer education (Powell et al. 2025). Forensically clean cultivated meat is a necessity, and regulatory agencies are working on microbial contamination risk assessment, genetic engineering issues, and growth factor usage, and it is essential to risk assess contaminant‐free meat (Post et al. 2020). Regulations on labeling are important to convey the quality of cultivated meat, its ingredients, nutrition value, and methods of production (Verbeke, Sans, and Van Loo 2015). Consumer education is vital to eliminate misconceptions and panic around cultivated meat consumption (Figure 3) (Bryant and Barnett 2018).

In the United States, the approval and regulation of cultivated meat is splintered between the Food and Drug Administration (FDA) and the United States Department of Agriculture (USDA) (Benson and Joel 2023). In the EU, cultivated meat is the responsibility of the EFSA, which reviews the safety of so‐called novel foods, which includes laboratory‐grown meat (Tuomisto and de Mattos 2011). To date, Singapore, Israel, and Canada have developed regulations around the production of cultivated meat (Stephens et al. 2018). These variances represent the different methods employed by different jurisdictions. For the purpose of foreign commerce and increasing consumer confidence, establishing a common regulatory framework is vital. Some international bodies, like the Codex Alimentarius Commission, pursue regional legislation integration (Post et al. 2020).

Across major jurisdictions, regulatory approval for cultivated meat is converging toward a broadly similar evidence package. Current frameworks typically require compositional analyses—including proximate nutrients, amino acid and lipid profiles, micronutrient levels, and screening for potential contaminants. In addition, agencies request toxicological and allergenicity data, batch consistency results, and detailed characterization of cell lines, growth media components, and bioreactor controls (EFSA Panel on Dietetic Products et al. 2021).

In the United States, the FDA's premarket consultation process further emphasizes documentation of cell bank establishment, manufacturing‐process controls, and hazard analyses aligned with the Federal Food, Drug, and Cosmetic Act (U.S. Food and Drug Administration 2023). The SFA includes additional expectations such as validation of biological substances in culture media, permissions for sensory evaluation, and real‐time monitoring plans for early pilot‐scale operations (Singapore Food Agency 2025). Taken together, these requirements illustrate the gradual emergence of a multicomponent safety dossier, broadly comparable to those used for novel foods and biotechnology‐derived ingredients internationally (EFSA Panel on Dietetic Products et al. 2016).

Beyond these common requirements, the use of genetically engineered (GE) cell lines introduces further regulatory considerations. Several authorities, including the EFSA, request extended molecular characterization data, genomic stability assessments, and, in some cases, GMO‐related labeling when GE modifications are incorporated into the production process (EFSA Panel on Dietetic Products et al. 2016). In the United States, GE‐modified cell lines fall under the FDA's biotechnology‐derived food oversight, which requires additional documentation regarding potential off‐target effects and the safety of introduced traits (U.S. Food and Drug Administration 2023). From a consumer acceptance perspective, studies in food biotechnology consistently show heightened risk perception toward GE‐associated products, indicating that transparency and effective communication may be important for maintaining willingness to try and willingness to pay (Siegrist and Hartmann 2020).

These regulatory differences also shape commercialization timelines, which vary markedly across regions. In the EU, the EFSA's scientific risk assessment phase can take up to 9 months, and subsequent Commission and Member‐State procedures commonly extend the full authorization process to approximately 2.5–3 years (EFSA Panel on Dietetic Products et al. 2021). Singapore, by contrast, has established a more streamlined approval pathway through the SFA, enabling early cultivated meat authorizations within roughly 18–24 months, supported by structured pre‐submission engagement mechanisms such as the Novel Food Virtual Clinic (Singapore Food Agency 2025). In the United States, the joint FDA–USDA framework has allowed companies to progress from premarket consultation to facility inspection and label approval within approximately 12–18 months, depending on manufacturing complexity (Benson and Joel 2023). These jurisdictional differences influence where companies initiate pilot launches, target early commercialization, and pursue export opportunities.

### 3.3 Leading Firms and New Businesses: An Evolving Innovation Ecosystem

Memphis Meats started as a single‐cell cultivation business and later widened its scope, changing its name to Upside Foods (Sim 2021). It now works with industry leaders like Mosa Meat, Aleph Farms, and Eat Just to create an integrated supply chain for cultivated meat (Bodiou et al. 2020). These startups have received substantial funding from giant players in the food sector, which has fostered the advancement of the technology and its commercialization.

There is active development of various types of bioreactors, such as scaffolding systems and advanced cell lines (Naing and Williams 2011). Because of this, the sustainable meat industry is undergoing rapid innovation as new companies seek to mimic traditional meat textures and tastes (Post et al. 2020). The industry has moved beyond cell cultures to multicellular meat construction, blended products, and novel processing techniques aimed at improving the marketing value (Bryant and Barnett 2018).

In the cultivated meat industry, collaboration and partnerships have become commonplace. Incumbent food companies are joining forces with other, more agile firms to brand new technologies with their know‐how (Specht et al. 2018). Such cooperation is needed to overcome technological hurdles, lower expenses, and widen the market outreach (Ching et al. 2022). With innovation at its core, the cultivated meat industry seeks to reengineer meat production to make it more sustainable, efficient, and ethical (“A Conversation About Cultivated Meat,” 2023).

Recent developments also reflect increased volatility within the cultivated meat industry. Several companies have undergone restructuring or workforce reductions due to capital constraints and scale‐up challenges, and a number of highly funded startups have reported delays or market exits (Watson 2025). For example, widely publicized reports described the shutdown of Believer Meats following financial and construction‐related difficulties (Ashkenazi 2025). These trends underscore the capital‐intensive nature of cultivated meat production and highlight the need for robust techno‐economic strategies and sustainable investment models.

## 4 Technological and Social Challenges in Scaling Cultivated Meat

### 4.1 Bridging Affordability and Scalability: Overcoming Technological Boundaries on Cultivated Meat Production

Despite recent progress, key technological gaps still hinder the widespread adoption of cultivated meat. Among them, the development of cost‐effective and scalable growth media remains a top priority. While earlier efforts have focused on replacing expensive animal‐derived components, emerging research now explores nutrient‐optimized, plant‐based formulations that also support large‐scale bioprocessing without compromising cell performance (Ianovici et al. 2022).

Another significant obstacle relates to the design of scalable bioreactor systems. Moving from small‐scale production to medium‐scale industrial production increases the production capacity, but requires a switch from small‐scale laboratory bioreactors to large‐scale industrial systems—all of which pose significant engineering difficulties (Bodiou et al. 2020; Moritz et al. 2015). New automated control systems and other designs for large‐scale productions are being developed to address the efficiency and cost effectiveness in order to meet the required demand (Bomkamp et al. 2022; Post et al. 2020).

Moreover, the production of cultivated meat with intricate musculature texture demands careful management of cell growth and tissue engineering. For cultivated meat to be feasible in the market, it has to replicate conventional meat's structure and texture (MacQueen et al. 2019). Researchers are optimizing scaffold‐based tissue engineering to better control cell differentiation to produce structured and edible meat products (Ben‐Arye and Levenberg 2019). Improving the efficiency of cell proliferation is also vital for shortening the production time and costs. Optimizing growth conditions and using genetic engineering are some of the more active endeavors (Ching et al. 2022; Rubio et al. 2020).

Overcoming these constraints requires a considerable amount of funding for research and development. A multidisciplinary approach involving scientists, engineers, and business professionals will be necessary to solve these issues and promote progress in the production of cultivated meat (Stephens et al. 2018).

Fabrication technologies play a central role in shaping the structure and sensory properties of cultivated meat. High‐moisture extrusion (HME) enables the formation of anisotropic, fiber‐aligned matrices that approximate whole‐cut textures, particularly in hybrid systems combining cultured cells with plant‐based protein scaffolds. Extrusion parameters—such as moisture content, screw speed, barrel temperature, and die geometry—directly modulate fiber formation and texturization (Mateen et al. 2023). Separately, three‐dimensional (3D) (bio)printing allows the spatial arrangement of myogenic and adipogenic cell‐laden bioinks within edible scaffolds, enabling precise control of tissue architecture and layered muscle–fat organization. Recent work highlights how optimized printing parameters and post‐printing maturation can improve structural fidelity and viability, offering a promising avenue for next‐generation cultivated meat fabrication (Ng et al. 2025).

Together, these fabrication technologies complement bioprocess optimization efforts by enabling cultivated meat to more closely reproduce the structural complexity and sensory characteristics of conventional whole‐muscle cuts.

### 4.2 Strategies of Change in Consumer Perspective: Moving Into Acceptance and Addressing Reluctance

Consumer perception of meat and meat products is based on dynamic psychosocial constructs; however, some commonly used quality cues include the brand and meat itself, along with taste and safety (Bryant and Barnett 2018). Reluctance due to fear can impede acceptance, which makes trust‐enhancing strategic communication necessary for diffusion. Those consumers who care about ethical and environmental issues may be more ready to accept cultivated meat if they realize its benefits for sustainability and animal welfare (Wilks and Phillips 2017). The reduced environmental harm, improved animal welfare, and antibiotic‐free production are great selling propositions (Tuomisto and de Mattos 2011). Herein, this study explores consumer perceptions of cultured meat and the importance of naturalness, directly addressing consumer reluctance (Onwezen et al. 2021). It also investigates the psychological factors influencing consumer acceptance of cultured meat, highlighting the significance of communication strategies.

Empirical studies indicate that perceived naturalness and safety strongly influence consumer willingness to pay (WTP) for cultivated meat. In a cross‐sectional experimental valuation study, respondents were willing to pay more for cultivated meat products when presented with favorable framing regarding environmental benefits and equivalent taste, but WTP decreased substantially when naturalness concerns were emphasized (Kantor and Kantor 2021). Additional segmentation research shows that distrust in technological food production and elevated risk perception can reduce WTP by 20%–40%, even among consumers initially open to alternative proteins (The Good Food Institute 2023). These findings demonstrate that WTP is highly sensitive to framing, familiarity, and safety perception, underscoring the need for transparent messaging and trust‐building strategies to support market adoption of cultivated meat.

One central hurdle is dealing with the negative “cultivated meat is unnatural” attitude. Fear can be reduced by educating consumers on the production process through clear communication (Verbeke, Sans, and Van Loo 2015). Confidence in the flavor and texture of cultivated meat can also be increased by showing it in natural settings, such as restaurants during taste trials, cooking shows, or even with sensory evaluation (Bryant and Barnett 2018).

Marketing strategies must focus on establishing a brand's credibility rather than selling aggressively. Messaging aimed at the public should elaborate on the safety procedures, safety oversight regulations, and nutritional value to assure consumer confidence in cultivated meat (Siegrist and Hartmann 2020). Social media, blogs, and other web‐based technologies can be paramount in enabling dialogue with the public, addressing their needs, and facilitating social acceptance (Apostolidis and McLeay 2016; Rolland et al. 2020).

Machine learning (ML) has emerged as a powerful tool for optimizing cultivated meat production. ML‐driven media optimization, for example, can reduce experimental burden and identify cost‐efficient formulations through predictive modeling. Advanced bioreactor control strategies employing soft sensors enable real‐time estimation of cell density, metabolite shifts, and contamination events (Ng and Tan 2025). In parallel, ML‐assisted scaffold and bioink design enhance printability and structural accuracy in 3D bioprinting workflows, while computer‐vision–based quality monitoring supports batch‐level consistency (Todhunter et al. 2024). Collectively, these applications highlight the growing role of ML in improving scalability, efficiency, and safety within cultivated meat systems.

### 4.3 Sustainability and Ethical Considerations: Stewards of Responsible Development and Long‐Term Optimism

Compared to other livestock systems, cultivated meat appears to be more ethical from an animal welfare and sustainability viewpoint. Nonetheless, its environmental impacts still need to be evaluated to ascertain whether its use is more sustainable than conventional meat (Tuomisto and de Mattos 2011). Life cycle assessments (LCAs) are useful instruments to assess the environmental impacts of cultivated meat, such as energy, water, and materials consumption, as well as the generation of different types of pollutants (Mattick et al. 2015). Such information is critical to design measures to make more sustainable production processes and manage possible negative impacts (Alexander et al. 2016).

Quantitative analyses from LCA studies provide clearer benchmarks for the environmental performance of cultivated meat relative to conventional livestock systems. (Tuomisto and de Mattos 2011) report that cultured beef has the potential to reduce land use by approximately 99%, water use by 82%–96%, and greenhouse‐gas emissions by 78%–96% compared with traditional European beef production (Tuomisto and de Mattos 2011). More recently, commercial‐scale projections by Sinke et al. (2023) indicate that cultivated meat produced using a low‐carbon (renewable) energy mix could achieve a 40%–80% lower global warming potential (carbon footprint) than conventional beef and pork systems. On the nutritional side, compositional studies show that prototype cultivated muscle tissues exhibit essential‐amino acid profiles comparable to poultry and beef, while allowing targeted modification of lipid classes—particularly increasing unsaturated fat ratios—relative to traditional meats (Fraeye et al. 2020). These quantitative comparisons reinforce claims regarding cultivated meat's environmental and nutritional potential, while also highlighting the dependence of outcomes on manufacturing scale and energy infrastructure.

Despite these potential benefits, several studies caution that large‐scale adoption of cultivated meat could introduce unintended environmental and social consequences. Energy demand for bioreactor operation may exceed that of poultry or pork production if renewable‐energy adoption remains limited, as highlighted by anticipatory life‐cycle analyses (Mattick et al. 2015).

Sector‐level transitions could also create socioeconomic disruptions, particularly in regions economically dependent on livestock production, where rapid shifts could widen rural–urban disparities (Rischer et al. 2020).

Equity concerns have likewise been raised, as high initial production costs may restrict access to affluent consumers, potentially exacerbating nutrition inequalities unless pricing and distribution models explicitly prioritize affordability (Hocquette 2016).

These considerations highlight the importance of pairing technological progress with decarbonization strategies, socially inclusive transition policies, and distributed manufacturing approaches to mitigate negative externalities (Sinke et al. 2023).

Along with these issues, the ethical debate must include animals' rights and food justice. Although cultivated meat does not require extensive animal husbandry and slaughtering, there are still concerns about the procurement of cell sources and their production's alignment with sustainability objectives. Moreover, equity in access to and affordability of cultivated meat is necessary to work toward global hunger alleviation and food justice (Hocquette 2016). If cultivated meat is left as a product for only the rich, its positive impacts would be greatly reduced.

Successfully producing cultivated meat long‐term relies on possible solutions to sustainability and ethical issues. If resolved, the sector could transform into a more economically viable and environmentally sustainable option than traditional meat, aiding in global food security and nutrition for a growing populace (Ben‐Arye and Levenberg 2019; Post et al. 2020).

## 5 Conclusion and Future Directions: Anticipating the Future of Food Through Cultivated Meat

Developed meat technology offers an effective answer to significant food industry problems related to sustainable practices and ethical safety standards. Recent advancements have occurred despite ongoing obstacles in technology, scalability, and production costs within the cultivated meat industry (Post et al. 2020). These advancements have drawn increased focus toward food production methods that uphold ethical standards and sustainability while ensuring safety. The path forward requires sustained research and development efforts combined with open stakeholder dialogue to conquer the current limitations of cultivated meat (Stephens et al. 2018).

Despite growing interest in cultivated meat, the evidence base remains constrained by several important limitations in existing studies. Most experimental work is conducted using small‐scale laboratory systems, making it difficult to extrapolate cell growth performance, nutrient profiles, or safety outcomes to industrial conditions. Nutritional data often originate from early prototypes that lack mature myofiber development, limiting the ability to draw firm conclusions about protein quality, lipid behavior, or micronutrient retention. Similarly, long‐term toxicological or metabolic data remain scarce, and current assessments of environmental impact or production costs depend heavily on modeled assumptions rather than validated industrial datasets. Consumer acceptance studies also largely rely on hypothetical products, which may not fully predict real‐world behavior. These gaps underscore the need for integrated biological, engineering, regulatory, and socioeconomic research efforts to build a more comprehensive evidence base.

Cultivated meat remains in its early technological stages, facing regulatory and technical hurdles that must be addressed before it becomes a viable mainstream food source. This article has explored the existing challenges and opportunities surrounding cultivated meat, particularly regarding nutrition, safety, economic feasibility, and legal considerations, while also addressing the social concerns related to food technology (Bryant and Barnett 2018). With respect to nutrition, one of the most promising aspects of cultivated meat is its potential to equal or surpass that of traditional meat. Novel methods of protein creation, nutrient supplementation, fat modification, and custom lipid profiles all have great potential for the enhancement of human health (MacQueen et al. 2019). Improvements in cell culture techniques and production systems, besides affordable growth media, will be critical in satisfying consumers' desires while enabling scalable production of cultivated meat (Humbird 2021). In addition, marketing strategies, as well as clear and easy‐to‐understand labels, will help build trust among consumers and guarantee adherence to regulatory norms (Rolland et al. 2020). Clear regulatory systems will provide assurance as well as encourage innovation so that trust in cultivated meat will develop alongside its use in the global food economy (Siegrist and Hartmann 2020).

The success of cultivated meat hinges heavily upon the ethical and sustainability ramifications. Justifiable procurement of cell lines, investments in animal welfare, and comprehensive life cycle assessments will be essential to validate its claimed environmental benefits (Tuomisto and de Mattos 2011). When the industry develops, the need for collaboration will change and there will be additional public issues that need to be dealt with to ensure that cultivated meat is produced sustainably (Wilks and Phillips 2017). Additionally, changing consumers' negative perception will require education and sensory marketing of cultivated meat and its defensive advantages in terms of taste, nutrition, and production (Verbeke, Sans, and Van Loo 2015).

As of now, the cultivated meat sector remains largely in a proof‐of‐concept and hybrid‐product phase, with most available items relying heavily on plant‐based scaffolds and incorporating cultured cells in minimal quantities (Alam et al. 2024). While this strategy facilitates earlier market entry and cost control, it raises concerns about consumer expectations, transparency, and nutritional equivalence (Treich 2021).

Thus, future advancements should prioritize the development of fully cellular, structured meat analogs, while ensuring clear labeling and honest communication about the composition of products. Only by aligning technological achievements with consumer trust and nutritional validity can the cultivated meat industry fulfill its promise as a truly transformative solution (Nurul Alam et al. 2024).

### 5.1 Future Research Directions

Several key research areas must be prioritized to accelerate cultivated meat's commercial viability. First, optimizing cell culture growth rates and improving cell viability through cost‐effective bioreactors and integrated design systems will enhance production efficiency (Bodiou et al. 2020). Achieving a scalable and economically viable process is critical for widespread market adoption. Another fundamental research area is the development of sustainable and affordable growth media. Current formulations rely heavily on animal‐derived components, leading to high costs and limited scalability. Investigating plant‐based or synthetic alternatives that maintain cell growth efficiency while reducing environmental impact will be crucial for cultivated meat's success. Moreover, refining cell differentiation and tissue engineering techniques is essential for achieving the texture, taste, and overall sensory experience of traditional meat. Advanced tissue engineering approaches, including scaffold development and bioprinting, will play a pivotal role in replicating the fibrous structure of conventional meat (Ben‐Arye and Levenberg 2019). These improvements will enhance consumer acceptance and market competitiveness. For cultivated meat to fulfill its potential, collaboration among scientists, policymakers, and industry leaders is imperative. By overcoming technical, economic, and regulatory barriers, cultivated meat can become a cornerstone of a sustainable and ethical food system, addressing global food security and environmental concerns (Post et al. 2020).

To realize the vision of cultivated meat, more attention is needed in certain areas. Improvement of cultured meat production technology can be a game changer for the industry, as these processes can be made highly efficient. This starts with managing the cell culture's growth rate and viability augmentation together with employing cheaper bioreactors, growth media, and integrative design systems. Development of such technology will enable the commercial production of cultivated meat. Another area deserving equal attention is the formulation of economically feasible and environmentally sustainable media. The use of animal‐derived constituents is too expensive, not scalable, and thus unsatisfactory. Research is needed to formulate plant‐derived constituent sources, which are affordable and eco‐friendly. Although these nutrients can single‐handedly improve the standing of cultivated meat in the marketplace, using these ingredients will make the product a more attractive choice, thus increasing the competitiveness of cultivated meat.

A crucial aspect of advancing cultivated meat technology is the ability to guide cell differentiation and engineer tissues that closely mimic the texture and sensory qualities of conventional meat. Accomplishing such a goal requires creating something completely novel that would encourage the cells to take on differentiation pathways that would, at the very least, create a product with meat‐like features. Achieving that level of complexity will undoubtedly depend on adequately advanced methods of tissue engineering. Realistic achievement of such types of meat requires an integrated effort by scientists from different disciplines, politicians, and business leaders. After the technical parameters, economic regulations, and legal barriers are dealt with, cultivated meat can become one of the significant components of a responsible and sustainable food system, addressing the problems of food deficiency and environmental sustainability.

## Author Contributions

**Sungmin Kim:** conceptualization, writing – original draft, writing – review and editing. **Keon‐Ha Park:** writing – review and editing, visualization. **Seong‐Jae Park:** writing – review and editing, visualization. **Yun‐Gwi Park:** writing – review and editing, visualization. **Sung‐Hwan Moon:** conceptualization, funding acquisition, supervision.

## Funding

This work was supported by the Bio&Medical Technology Development Program of the National Research Foundation (NRF) [No. RS‐2023‐00220207], the Korean Fund for Regenerative Medicine (KFRM) grant funded by the Ministry of Science and ICT, the Ministry of Health & Welfare [25A0203L1], Korea government, and the Chung‐Ang University Graduate Research Scholarship (Academic scholarship for College of Biotechnology and Natural Resources) in 2025.

## Ethics Statement

Sungmin Kim, Keon‐Ha Park, Seong‐Jae Park, Yun‐Gwi Park, and Sung‐Hwan Moon declare that they have no conflicts of interest. This article does not contain any studies with human or animal subjects performed by any of the authors.

## Conflicts of Interest

The authors declare no conflicts of interest.

## Data Availability

Associated data is completely presented in this manuscript.
